# Book Reviews

**Published:** 1830-06

**Authors:** 


### CRITICAL ANALYSES.

Quæ laudanda forent, et quæ culpanda, vicissim

Illa, prius, cretâ; mox hæc, carbone, *notamus*.—Persius.

### *A Treatise on Poisons, in relation to Medical Jurisprudence, Physiology, and the Practice of Physic.* By Robert Christison, M.D. &c. &c.

In our Number for February, we analysed and reviewed the general doctrines of toxicology contained in Professor Christison's work; and we now proceed to present our readers with an account of the manner in which he has treated of poisons individually. In executing our purpose, we shall follow the order traced out by the author, with the exception that we shall exclude the chapter on arsenic, as the facts and experiments on that subject detailed in the present work must be already familiar to medical men in general, through the excellent essays formerly published by the author.

I. And first of the Irritant Poisons, which are divided into five orders : the acids and their bases; the alkalies and their salts; metallic compounds; vegetable and animal irritants; mechanical irritants. In discussing the effects of the mineral acids, our author is largely indebted to the excellent memoir of Tartra.* Poisoning by the mineral acids has been chiefly the result of suicide or accident: instances of criminal poisoning by the mineral acids have, however, occurred, and of late years "the crime of disfiguring the countenance, by squirting oil of vitriol on it," has arisen, and is made by law a capital offence.

"M. Tartra considers that four varieties may be observed in the effects of nitric acid and other mineral acids: 1. Speedy death, from violent corrosion and inflammation; 2. Slow death, from a peculiar organic disease of the stomach and intestines; 3. Imperfect recovery, the person remaining liable ever after to irritability of the stomach; 4. Perfect recovery."

We shall speak only of the immediate and violent consequences of the mineral acids: these are, symptoms of violent gastritis, accompanied with burning of the throat, eructations, and an excruciating pain of the stomach. The lips are commonly shrivelled; at first whitish; afterwards, if from nitric acid, yellowish; if from sulphuric acid, brownish. The inside of the mouth is also generally shrivelled,

* Traité de l'Empoisonnement par l'Acide Nitrique. 1802. Paris.

white, and often more or less corroded; and, as the poisoning advances, the teeth become loose and yellowish brown. This discoloration has taken place in so short a time as three hours. The matter vomited is generally brownish or black, and causes effervescence on the pavement, if it contains lime. Afterwards this matter is mixed with shreds of membrane, which sometimes actually consist of the disorganized coats of the stomach, but are generally only coagulated mucus. The bowels are obstinately costive, the urine scanty or oppressed; and the patient is frequently harassed by distressing tenesmus and desire to pass water. The pulse is all along very weak, and towards the close imperceptible, sometimes intermitting. It is not always frequent; on the contrary, it has been observed of natural frequency, small and feeble, in a patient who survived fifteen days. The countenance becomes at an early period glazed, and the extremities cold and clammy. The breathing is often laborious, the movements of the chest increasing the pain in the stomach, independently of the pulmonary inflammation which is also at times present. To these symptoms are added occasional fits of suffocation, from the shreds of thick mucus sticking in the throat.

Such are the *usual* symptoms, but it must not be supposed that they are always so well developed. The duration of this kind of poisoning is commonly between half a day and two or three days; but the fatal event has taken place in very few hours, or it may be deferred for many days. The smallest fatal dose of sulphuric acid recorded was one drachm; a man has recovered after taking six drachms.

Death may result from these poisons, Dr. Christison hints, from their effects on the gullet producing dysphagia, or by exciting inflammation and spasm of the glottis and larynx.

In the treatment of this form of poisoning no delay is allowable. Chalk or magnesia are the antidotes which ought to be preferred; but, if it will consume time to procure them, a solution of soap should be administered, and, during its preparation, dilution with milk or oleaginous matters should be used. The carbonates of the alkalies are ineligible. After the proper antidote has been given to a sufficient extent, the use of diluents ought to be continued, as they facilitate the vomiting. Subsequently, the usual remedies for inflammation may be requisite.

We pass over the chemical analysis (pp. 120 et seq.) which, however, display the author's usual talent and delicacy of manipulation. The processes for detecting sulphuric acid are easily performed, and are decisive: those for nitric and muriatic acids are not so satisfactory.

The chapter on phosphorus, sulphur, chlorine, and iodine, calls for no particular observation from us. The essay of Dr. Gairdner contains an excellent case, illustrative of the irritant effects of large medicinal doses of iodine.

Oxalic acid is the last poison of the present order, but its effects and antidotes are so well known as to require but little remark. One attempt has been made to make it an instrument of murder.

Instances of death from this acid have, it is well known, almost invariably arisen from the accidental substitution of it for the sulphate of magnesia. So close, indeed, is the resemblance of these two salts, that "repeatedly," says Dr. C., "in desiring several persons (qualified to form a probable judgment, of course,) to point out which was the poison and which the laxative, I have found as many fix on the wrong as on the right parcel. The sulphate of magnesia has, of course, a very different taste, being strongly bitter. Various plans have been devised for preventing the accidents to which this unlucky resemblance has given rise. The best of them imply the use of a criterion or safeguard by the patient before he takes his laxative draught. It seems to have escaped the notice of those who have proposed the plans in question, that, if accidents are to be prevented in this manner, by far the simplest and most effectual security will be to let the public know that a laxative salt ought always to be tasted before it is swallowed."

The proper antidotes for oxalic acid are chalk or magnesia, with which insoluble oxalates are formed. The soluble oxalates of potass, &c. are nearly as poisonous as the acid itself. Diluents should not be used unless the vomiting is free, as they favor the absorption of the acid. If (after poisoning by a concentrated solution of oxalic acid,) the stomach is examined immediately after death, little corrosion will be found, compared with what is seen if the inspection be delayed a day or two.

In instancing the periods within which oxalic acid has proved fatal, Dr. Christison has quoted a case* by Mr. Frazer, which he describes as having terminated fatally at the end of twenty-three days; but this is a mistake, the patient having survived but fourteen days. He swallowed

* Edinb. Med. and Surg. Journal, xiv. 606.

the poison on the 16th of the month, (it is printed the 6th, hence the mistake,) and died on the 29th. It has proved fatal in ten minutes, and repeatedly within an hour.*

2. The alkalies and alkaline salts are, for the most part, local irritants. Some of them likewise act indirectly on distant organs, and a few are more distinguished by their remote than by their local effects. Dr. Christison divides them into two groups, the one embracing the two fixed alkalies, with their carbonates and nitrates, and also lime; the other ammonia, with its salts, and likewise the alkaline sulphurets.

"In the treatment of poisoning with the alkalies, the first object is evidently to neutralize the poison. This may be done either with a weak acid or with oil." The acetic is to be preferred. "Very lately, however, a French physician, M. Chereau, has stated that, for the mineral alkalies and their carbonates, fixed oil is a preferable antidote; and he has given the heads of two cases of poisoning

* We are indebted to a friend for the two following cases of poisoning with oxalic acid.

A farmer, in August 1825, took a quantity of oxalic acid (supposed to be an ounce,) in mistake for Epsom salts. He was seen in about an hour afterwards. He complained of burning heat in the mouth, throat, and stomach; great thirst, and inclination to vomit. The tongue was swollen; the countenance betrayed great anxiety; the pulse small, quick, and irregular; and his appearance showing great suffering and distress. Chalk and milk were given in large quantities, and in about ten minutes the pain and heat began to abate. An emetic was then administered, and afterwards repeated, so as to wash out the stomach, as it were, for nearly two hours, until all pain and heat had nearly subsided. He continued the chalk and milk for two or three days; and, with the use of mild purgatives, gradually recovered in three or four weeks.

" A butcher, in April 1829, took a quantity of oxalic acid (supposed ℥ss.) intentionally. He was visited in fifteen or twenty minutes. He had been previously drinking, and was found nearly in a state of insensibility, as was supposed from that cause. The pulse was so small as scarcely to be felt, tongue rather swelled, and the eyes fixed. With some difficulty, chalk and water was administered, and afterwards an emetic, the operation of which roused him from his stupor. The chalk and water were continued; and the next morning he was able to go out and follow his business.

The former of these cases, if compared with the one related by Mr. Frazer (Edinb. Med. and Surg. Journal, xiv.) will show the superior neutralizing power of the chalk over magnesia, or at least the comparatively innocuous power of the compound thus formed. Mr. Frazer's patient took half an ounce, and the magnesia was freely administered in something more than forty minutes, yet he lost his life, from the disorganization of the villous coat of the stomach, &c., after fourteen days. In the case above quoted, an ounce was swallowed, the remedy was not administered for an hour, yet the relief was decided and permanent; for, although the man suffered from stomach complaints for more than three weeks, we understand the symptoms were such as to cause no anxiety for the result.

In the latter of the two cases, the antidote, administered in about twenty minutes, was so efficacious that the man was enabled to enter on a considerable journey on the following morning.

with large doses of carbonate of potass, in which the free employment of almond oil prevented the usual fatal consequences. It appears to act partly by rendering the vomiting free and easy, partly by converting the alkali into a soap. It must be given in large quantity, several pounds being commonly required." (P. 159.) An interesting case of accidental poisoning by American potass in a state of deliquescence, successfully treated by Mr. CUMIN, of Glasgow, is related in the last volume of the Edinburgh Med.-Chirurgical Transactions.

Cases of accidental death have occurred from swallowing Lime, but it is a feeble poison. "When thrown into the eyes, it causes acute and obstinate ophthalmia, which may end in loss of sight: on this account it will belong, I presume, to the poisons included in the Scottish act against disfiguring or maiming with corrosives."

Some years since we saw a case, which, under a less fortunate termination, might have become the subject of judicial investigation. A man had a large sarcomatous tumor situated at the back of the neck, which, if removed, would have weighed probably two pounds. A quack, to whom he applied, recommended the application of quick lime to the whole tumor. Extensive inflammation and ulceration followed the application; the ulcers were very ill conditioned, and attended with fever and gradual emaciation. For more than a year the man's recovery was doubtful; eventually, however, he did recover, the tumor having at length entirely disappeared.

Under the head of Ammonia, three cases of violent inflammation of the mucous membrane of the air-passages, from the excessive *inhalation* of ammonia, are described. Two of these proved fatal; and M. NYSTEN has recorded the morbid appearances discovered in one: "The nostrils were blocked up with an albuminous membrane; the whole mucous membrane of the larynx, trachea, bronchi, and even some of the bronchial ramifications, was mottled with patches of lymph; the gullet and stomach showed red streaks here and there." (P. 168.) It is worthy of observation, that the remote nervous symptoms which sometimes occur from ammonia, when taken in poisonous doses, were not observed in these cases.

Four cases of poisoning in the human subject by liver of sulphur (sulphuret of potass) have been related. "Of these cases two have proved fatal, both in less than fifteen minutes, and the symptoms preceding death were acrid taste, slight vomiting, mortal faintness, convulsions, and a striking chemical sign, the tainting of the air of the chamber with the odour of sulphuretted hydrogen. The dose in one case was about three drachms." (P. 171.) These observations manifest the impropriety of employing this substance as a remedy in poisoning by arsenic, which has been done occasionally.

In this kind of poisoning, the most appropriate treatment "seems to consist in the instant administration of any diluent which is at hand, the subsequent exhibition of frequent doses of the chloride of soda, (common salt,) and then the antiphlogistic mode of subduing inflammation. The chloride of soda or lime may be called the antidote against this poison, as it decomposes the sulphuretted hydrogen which is evolved, and the rapid disengagement of which is the probable cause of death in the quickly fatal cases. The symptoms, at least, are very nearly those of poisoning with sulphuretted hydrogen when introduced into the system in a more direct manner." (P. 171.)

The preparations of *mercury* have been often employed for criminal purposes, and thus become the subject of judicial inquiry.

"In another respect, too, they claim the regard of the medical jurist: their effects on the body when insidiously introduced in the practice of the arts in which mercury is used, form a branch of that department of medical police which treats of the influence of trades on the health." (P. 268.)

The chapter on Mercury is very valuable. The chemical details are ingenious, and the modes of analysis recommended simple, decisive, and easily executed.

*On the mode of action of mercury, and the symptoms it excites in man.* The effects of mercury on the animal body are more diversified than those of any other poison; but its various compounds have not all been experimentally examined with equal care: the more immediate and prominent properties of corrosive sublimate have been chiefly elucidated; and Dr. Christison states, as the result of the inquiry, "that corrosive sublimate causes, when swallowed, corrosion of the stomach, and, in whatever way it obtains entrance into the body, irritation of that organ and of the rectum, inflammation of the lungs, depressed action, and perhaps also inflammation, of the heart, oppression of the functions of the brain, inflammation of the salivary glands." (P. 289.) But in man other organs still are implicated.

Dr. Christison notices at some length the controversy connected with its mode of action, namely, whether, before it can exert its remote action, it must enter the blood. "The chief facts in support of the affirmative may be arranged under three heads. Some relate to the discharge of metallic mercury from the living body during a mercurial course for medicinal purposes; others to the discovery of metallic mercury in the dead body, in the like circumstance; and others to the detection of mercury by chemical analysis in the fluids and solids, during life or after death." (P. 290.) The first series of facts is well illustrated by the quotations of Dr. Christison; and the second also by the satisfactory case of Rigby Brodbelt, (Mem. Lond. Med. Soc. v. 112,) which might be further supported by reference to the observations of some modern pathologists; for instance, Lobstein and Otto.* On the last head the facts are contradictory, but "they furnish at least a presumption that it is present in some circumstances in the animal fluids." (P. 293.) But the chemical investigation is attended with very considerable difficulty, and certainly the negative evidence from this source is any thing but decisive.

"The cases of poisoning with mercury, which have been observed in the human subject, may be conveniently arranged under three varieties. In one variety, the sole or leading symptoms are those of violent irritation of the alimentary canal. In another variety, the symptoms are at first the same as in the former, but subsequently become conjoined with salivation and inflammation of the mouth, or some of the other disorders which indicate mercurial erethism, as it is called. In a third variety, the preliminary stage of the last is wanting, and the symptoms are from the beginning those of mercurial erethism, in one or other of its multifarious forms.

"The first variety of poisoning with mercury is remarked only in those who have taken considerable doses of its soluble salts, particularly corrosive sublimate. The second is caused by the same preparations. The third may be caused by any of the compounds of mercury." (P. 294.)

In the first variety, the symptoms of irritation of the throat and stomach appear earlier than from arsenic; the taste (from corrosive sublimate) is more strong and unequivocal, and the sense of acridity is usually much stronger, and the burning tightness of the throat very considerable; the countenance is much flushed and swelled; painful micturition is also common, sometimes even ischuria renalis; and there is a good deal of uniformity in the symptoms from this poison. Dr. Christison states the ordinary duration in fatal cases from twenty-four hours to three days, the shortest eleven hours; but these limits he does not state with confidence.†

* Lehrbuch der Pathologischen Anatomie, u. s. w. Berlin, 1830, p. 157.

† We saw, some years ago, a case of poisoning with corrosive sublimate, which terminated fatally fourteen days after taking the poison. A young

Of the second variety of mercurial poisoning, Dr. Christison has quoted a striking example from the 41st volume of this Journal.

The third variety of poisoning with mercury comprehends all the forms of what is called mercurial erethism; and these are "most frequently the consequence of the milder compounds of mercury, either given medicinally in frequent small doses, or applied continuously to the bodies of workmen who are exposed by their trade to its fumes." (P. 301.) Dr. Christison's remarks apply to salivation and the *tremblement mercuriel*, or shaking palsy.

As the phenomena of mercurial salivation have been often known to yield important evidence, and have led to contrariety of opinion upon trials, they deserve particular attention.

Fifteen grains of bluepill, taken in three doses, one every night, have excited fatal salivation. Two grains of calomel have caused ptyalism, extensive ulceration of the throat, exfoliation of the lower jaw, and death. On the other hand, some constitutions and states of disease resist the action of mercurials very obstinately.

But salivation may be an idiopathic disease, or the effect of imagination, or produced by other substances than mercury, namely, preparations of gold,* or copper, antimony, iodine, croton oil, foxglove, and even opium; and, as the evidence of salivation may be quoted as proof of mercurial poisoning, it is important to ascertain the distinguishing signs of true mercurial ptyalism.

"In general, mercurial salivation may be easily distinguished from all the preceding varieties by an experienced practitioner... Its characters are quite distinct at the time salivation just begins: the fetor of the breath, and sponginess and ulceration of the gums at this stage, distinguish it from every other affection. But if the state of the mouth is not examined till the ulcers have existed sewoman, in the fourth month of pregnancy, swallowed a large dose of corrosive sublimate, certainly more than a drachm. An hour and a half elapsed before any antidote could be administered; during that time she had been sick, and the vomiting had been promoted. The free administration of white of egg relieved all the urgent symptoms, and it was hoped that the case would end well. During the two or three following days, the patient complained but little; she had, however, griping, with a good deal of pain in the rectum, and tenesmus. At the end of a week, moderate salivation came on; the bowel complaint rather increased; but the most conspicuous symptoms were low fever, with very dry skin and rapid emaciation. At the end of a fortnight she miscarried, and sunk in a few hours under the trifling hemorrhage which succeeded. Up to the time of abortion, she had been able to walk about and go out of doors.

* We last year saw, in the Hôpital Beaujon at Paris, a case of extensive ulceration of the throat, with loss of substance, following salivation produced by one of M. Chretien's preparations of gold.

veral days, the characters of the mercurial disorder are much more equivocal. They cannot be distinguished, for example, from some forms of idiopathic ulceration of the mouth connected with unsoundness of constitution, and characterized by extensive sloughing, ptyalism, and gangrenous fetor." (P. 306.)

But a long interval may elapse after the administration of the mercury has been abandoned, before the effect on the salivary organ begins; mercury in small doses being a "*cumulative* poison." Swédiaur has met with instances where the interval was several months; Cullerier, with a case in which it was three months. And, after the remission of the mercury, the ptyalism may continue for a long period: Linnæus met with an instance of its continuing inveterately for a whole year; and M. Colson knew an individual whose salivation continued during six years. After an ordinary mercurial course, the mouth and salivary glands generally return to the healthy state in the course of a fortnight or three weeks. Ptyalism, in some rare instances, may recur after a considerable interval; no more mercury having in the interim been administered. The testimony to this fact of Messrs. Bromfield and Howard, of the Lock Hospital, on the trial of Miss Butterfield, apparently led to her acquittal.

Death from salivation may result from extensively spreading gangrene; from exhaustion from profuse and protracted discharge of saliva; and Dr. Christison has seen it fatal by inducing laryngeal phthisis. (P. 309.)

Dr. Christison's remarks on *shaking palsy*, or *tremblement mercuriel,* are derived chiefly from Mérat, (Traité de la Colique Metallique.) In the eighth and ninth volumes of the Edinburgh Med. and Surg. Journal will be found some valuable remarks on this state by the late Dr. Bateman, which Dr. Christison has not noticed. According to Mérat (Christison, p. 311,) the tremors are cured easily, though slowly, some months being required. Dr. Bateman, on the contrary, concurs with Fernelius in considering them "tremores immedicabiles." At the expiration of several months, it is certain that the tremors in his patient were unaltered.

In vol. ix. of the London Med.-Chir. Transactions, Dr. Bateman has described his own case of mercurial erethism, in which violent and irregular action of the heart was induced, with sense of constriction in the region of the diaphragm. This case, it will be recollected, was of long continuance, and was successfully treated with stimulants. In vol. xiv. of the Edinburgh Med. and Surg. Journal, two analogous cases are related by Dr. Astbury. In these cases the muscles of respiration appear to have been partially paralysed. One terminated fatally from paralysis of the heart. In the year 1818, we had an opportunity of observing a similar case. A stout man, convalescent from a slight attack of inflammatory typhus, was taking purgatives with a little calomel each night, for four or five successive nights. At the end of this time, being pretty well and taking exercise, he complained of pain about the sciatic nerve of one side, with some inability in moving the affected limb. The eyes were heavy, watery, and a little suffused, and the breathing not quite free. His complaints, however, did not appear of importance: but, on the second night from this invasion, he became suddenly very ill; his breathing slow, laborious, and irregular, with rattling in the throat; the pulse not exceeding forty in a minute, and irregular; very profuse perspiration, with great prostration of strength. The mind was clear and tranquil. Stimulants were administered continually; and, if they were remitted for ten minutes, he appeared to be threatened with immediate cessation of breathing and of the heart's action. The doors and windows of the apartment were kept open, at the earnest request of the patient. During the night he took two bottles of wine, in addition to brandy and water, ammonia, and ether. The latter, with assafœtida mixture, appeared to produce the greatest relief. At the expiration of about eight hours, amendment was perceptible, and became progressive; and in a few days he had pretty much recovered. He has since enjoyed very good health. Dr. Bateman derived the greatest relief from musk. Mercury in its metallic state is probably inactive. "It has been said," says Dr. C. "that patients who have taken it for obstructed bowels have sometimes been salivated. This accident, though exceedingly rare, has nevertheless, I believe, happened in a few instances in which the mercury was retained long in the body. Fluid mercury, then, is probably not altogether inactive, speaking medico-legally. But this admission is no argument in favor of the metal being physiologically a poison; because, in the course of the cases referred to, a part is, in all likelihood, oxidated by the oxygen in the intestinal gases."

The *sulphurets* do not possess any deleterious action. The *red precipitate* and *turbith mineral* are irritants, and will produce erethism; but they are not escharotics, that is, they do not chemically corrode the animal textures. Of corrosive sublimate we have said enough. The nitrates, like the latter, are corrosive. The cyanide resembles corrosive sublimate, except that it does not chemically corrode.

Calomel is an irritant; but the practice followed by the East and West India practitioners of administering large doses in certain diseases, shows that it is not so under all circumstances. Probably it may be correct to transfer the reasoning of Rasori on the effects of emetic tartar to calomel, namely, that it operates on the animal economy very differently in a state of health and disease. Without doubt the impurity of the preparation may be an occasional source of mischief; and we submit that Mr. Howard's mode of preparing it should be always practised to ensure an uniform preparation. Dr. Paris, in his Pharmacology, observes that Howard's calomel is more active than that prepared according to the Pharmacopœia, probably from its state of minute division.

In poisoning by corrosive sublimate, we fortunately possess an antidote, which with regard to arsenic, is still a desideratum. Albumen and the gluten of wheat decompose corrosive sublimate. According to Peschier, the white of one egg is required to render four grains of the poison innocuous. "Albumen," says Dr. C. "is chiefly useful in the early stage of poisoning with corrosive sublimate, and is particularly called for when vomiting does not take place; but it further appears to be an excellent demulcent in the advanced stages." (P. 329.) In the case we have alluded to, p. 13, the white of egg was decidedly and immediately useful at the distance of an hour and a half after the swallowing of the poison. In this case some of the poison had probably already passed the pylorus, as salivation came on afterwards; but all the urgent symptoms were removed, nor did they recur. "When neither albumen nor gluten is at hand, milk is a convenient antidote of the same kind."

In mercurial salivation, it has been proposed by Dr. Finlay to check it by small doses of tartar emetic, frequently repeated, so as to act on the skin; and Mr. Daniell has recommended large doses of the acetate of lead as an effectual remedy for the same purpose. Dr. Christison has tried the former of these plans several times with apparent success. The chloruret of soda (as it has been called) has been recommended in gargle in salivation, we believe.

*Copper*. Natural verdigris (carbonate of copper) is insipid and quite insoluble, so that pure water left in vessels incrusted with it does not become poisonous. Artificial verdigris differs in composition according to the mode of manufacture. Foreign verdigris contains the neutral acetate, the subacetate, a little carbonate, oxide, and even metallic copper, along with particles of the fruit and fruitstalks of the grape. British verdigris consists chiefly of a mixture of the neutral acetate and subacetate, the former of which is soluble, and the latter insoluble in cold water. This difference enables the chemist to separate these substances from one another. If copper vessels be kept perfectly clean, they are not improper for culinary purposes. Some acid matters, however, though they do not dissolve clean copper by being merely boiled in it a few minutes, nevertheless, if allowed to cool and stand some time in it, will acquire a sensible impregnation. Dr. Christison has quoted an illustrative case from Wildberg. "A servant left some sour krout for only a couple of hours in a copper pan which had lost the tinning. Her mistress and a daughter, who took the cabbage at dinner, died of twelve hours' illness ; and Wildberg found the cabbage so strongly impregnated with copper, that it was detected by the test of metallic iron."

Vinegar dissolves metallic copper: and this depends on the cooperation of the atmospheric air held in solution by the fluid, and in contact with its surface; for, without such cooperation, the copper cannot be oxidated. Fatty matters and oils, particularly volatile oils, have the property of oxidating and uniting with copper.

"The symptoms caused by the soluble salts of copper in man are, in a general point of view, the same as those caused by arsenic and corrosive sublimate. According to the cases related by Orfila, the first symptom was violent headach, then vomiting and cutting pains in the bowels, and afterwards cramps in the legs and pains in the thighs. Sometimes, throughout the whole course of the symptoms, there is a peculiar coppery taste in the mouth, and singular aversion to the smell of copper. Drouard notices this in his thesis, and says that, having himself been once poisoned with verdigris, the smell of copper used for a long time after to excite nausea. Another symptom, which occasionally occurs in this kind of poisoning, and never, so far as I know, in poisoning with arsenic or corrosive sublimate, is jaundice. It likewise appears, when the case ends fatally, that convulsions and insensibility very generally precede death." (P. 349.)

A very small quantity of the sulphate of copper, says Dr. C., will prove fatal; for Drouard found that six grains killed a dog in half an hour. To this statement there are, however, exceptions. An old woman, addicted to intoxication, on being remonstrated with, swallowed a quantity of sulphate of copper, supposed to be about two drachms. She soon vomited. In an hour afterwards, when we saw her, she had severe pain in the stomach and head ; but the vomiting continued, and the following day she was nearly free from complaint. She had neither jaundice, convulsions, nor diarrhœa. Probably in M. Drouard's experiments the œsophagus was tied.

On this treatment in this variety of poisoning, it is proper to remark that sugar, which was formerly supposed to be an antidote, is not so; and that Orfila has found that albumen and ferro-cyanate of potass are the proper remedies. The former will doubtless be preferred, and should be given liberally, even after the urgent symptoms have disappeared.

We now pass on to the chapter on *Lead*, one of the most judicious and original in the volume; the subjects treated of in the intermediate pages not calling for any particular observation. Extending his researches beyond the limits of practical medicine and medical jurisprudence, our author has investigated the properties of lead in relation to medical police. We shall first speak of the tests of lead.

"In the fluid state, the acetate of lead, as well as all the soluble salts of lead, may be detected by the following system of reagents: Sulphuretted Hydrogen, Chromate of Potass, Hydriodate of Potass, and Metallic Zinc.

"1. *Sulphuretted hydrogen gas* causes a black precipitate, the sulphuret of lead. This is a test of extreme delicacy, and acts in whatever state of combination the lead exists, whether fluid or solid. It is preferable to the hydrosulphate of ammonia as a medico-legal test; for, as Fourcroy observed, the hydrosulphate of ammonia acts on many sound wines as if they contained lead, while the sulphuretted hydrogen never causes with them a black precipitate, unless they contain either lead or some other metallic impregnation. It must be remembered that many other metallic solutions yield a black precipitate with this test.

"2. *Chromate of potass*, both in a state of protochromate and bichromate, causes a fine gamboge-yellow precipitate, the chromate of lead. For the characteristic action of this reagent, it is desirable that the suspected liquid be neutral. It forms, with solutions of the sulphate of copper, a precipitate nearly of the same colour as the chromate of lead.

"2. *Hydriodate of potass* causes also a lively gamboge-yellow precipitate, the iodide of lead. The action of this test is impaired in delicacy by a considerable excess of nitric or acetic acid: these acids cause a yellow coloration with this test, although no lead be present.

"4. If the solution of lead is not too dilute, *a piece of zinc*, held for some time in it, displaces the lead, taking its place in the solution; and the lead is deposited in the form of a crystalline arborescence. This is a very characteristic and even delicate test. It acts also on the nitrate of lead." (P. 383.)

The value of the preceding tests is diminished or destroyed by the presence of animal or vegetable matters. The sulphuretted hydrogen undergoes the least alteration: this may be applied so as to detect lead in all possible states of mixture. But, before proceeding with this subject, Dr. Christison points out the various ways in which it is apt to be insidiously introduced into the body.

1. *Of the action of air and pure water on lead.* Lead is tarnished by exposure to air, by which the *carbonate* (not the oxide) is formed, and this is accelerated by the presence of moisture. Dr. Lambe, Guyton Morveau, and Dr. Thomson of Glasgow, have investigated the effects of water on lead. Dr. Christison has extended their researches, and, experimenting with his usual ingenuity and precision, has obtained the following results.

"The general result of these experiments appears to be, that neutral salts in various, and for the most part minute proportions, retard or prevent the corrosive action of water on lead, allowing the carbonate to deposit itself slowly, and to adhere with such firmness to the lead as not to be afterwards removable by moderate agitation; adding subsequently to this crust other insoluble salts of lead, the acids of which are derived from the neutral salts in solution, and thus at length forming a permanent and impermeable skreen, through which the action of the water cannot any longer be carried on." (P. 390.)

The presence of free carbonic acid, however, impairs the preservative power of the neutral salts.

*Of the action of natural waters on lead.* Rain or snow water collected at a distance from a town or buildings, is pure, and hence corrodes lead; but, collected in a town, its activity is much impaired, but still it will corrode. "Hence, perhaps, even in a town, but at all events certainly in the country, it would be wrong to use, for culinary purposes, rain or snow water which has run from lead roofs or spouts recently erected."

Spring waters have, *in general*, little or no action on lead, because they contain neutral salts. They do, however, sometimes corrode lead with rapidity, and thus lead to serious and fatal diseases. An instance of the kind is quoted from a paper by Dr. Wall, (Transactions of Lond. Coll. of Physicians, ii. 400.) The water in this instance Dr. Christison presumes must have contained either an unusually small proportion of salts, or a large proportion of carbonic acid. The Doctor illustrates "the danger of keeping the *same portion* of water for a length of time in leaden cisterns, if it has the power of acting on lead even in a trifling degree;" and, although it has been stated that air is necessary to the formation of the carbonate, that air may exist in the water, and hence affect close pipes. Gmelin observes that the solvent power of water on leaden pipes is much increased if the pipes have a considerable fall; which Dr. Christison explains by saying that it is because the protecting crust can never fairly form before it is swept away.

*On the action of acidulous fluids on lead, and on its oxide.* Water which is acidulated with various acids acts on lead, with different degrees of rapidity. Carbonic acid partially counteracts the preservative effects of the neutral salts. The vegetable act on lead more vivaciously than the mineral acids (diluted); the acetic most strongly, then the citric, and then the tartaric. Hence the preparation or preservation of articles of food and drink in leaden vessels is fraught with danger; and in this way lead has often been insidiously introduced into the food of man. Milk has been rendered poisonous by being kept in leaden vessels. Rum has been supposed to be similarly affected, but this is somewhat doubtful. Wine has been accidentally impregnated in consequence of the bottles having been cleaned by means of shot, and some of the shot left behind. Cyder, from the presence of lead in the apparatus of the cyder houses; the action of vegetable acids on the glazing of earthenware, which contains an oxide of lead.

But many articles are designedly adulterated with lead for a variety of purposes; and among these the most common is the addition of litharge, to correct acescency in wines. "It is probable, however," says Dr. Christison, "that the adulteration of wine with lead can only be practised with success on the common tart kinds, such as those used by the lower orders on the continent:" for, in fact, it is only *when the acescency is due to the presence of acetic acid that lead is at all useful.* In France, it is a common practice to clarify honey and sugar of grapes, and to make brandy pale, by means of sugar of lead; and M. Boudet has detected lead in many samples of these articles as sold in Paris. Cheese has been intentionally adulterated with red lead, in order to communicate the peculiar reddish-yellow colour which is supposed to be characteristic of fineness of quality.

The process for detecting lead in mixed fluids, is next described.

"The symptoms observed in man from the preparations of lead are of three kinds. One class of symptoms indicates inflammation of the alimentary canal; another, spasm of its muscles; and a third, injury of the nervous system, sometimes apoplexy, more commonly palsy, and that almost always partial and incomplete. Each of these classes of symptoms may exist independently of the other two; but the two last are more commonly combined." (P. 412.)

The morbid appearances caused by poisoning with lead are in some respects peculiar. The valuable work of Mérat contains an account of four inspections after death from the acute or comatose form of Colica Pictonum. The bodies were plump, muscular, and fat. The alimentary canal was quite empty, and the colon was much contracted, in one to an extraordinary degree. The mucous coat of the alimentary canal was everywhere healthy. No morbid appearance was visible within the head. The appearances in those who have been long affected with the paralytic form of colica pictonum have been rarely observed. Dr. Duncan supplied to Dr. Christison the only good account he has been able to procure of the inspection of the intestinal canal in such a case. The man, who was a plumber, had been long and frequently afflicted with colica pictonum and its sequelæ. The intestines were dark, tender, and far advanced in putrefaction; the cardiac orifice of the stomach was so narrow that it would only allow a goose-quill to pass; the mesenteric glands were enlarged and hardened; the thoracic duct was surrounded by many large bodies like diseased glands, exactly of the colour of lead, and composed of organised cysts, containing apparently an inorganic matter. The muscles in similar circumstances are much diseased. When the paralysis is not of long standing, it appears, from the experiments of Schloepfer, (whose animals survived about three weeks,) that the whole muscular system becomes pale, bloodless, and flaccid. When the palsy is of long standing, this change increases so much, that the muscles in some parts, as in the arms and thumbs, acquire the colour and general aspect of white fibrous tissue.

"In the irritant form of poisoning, a safe and effectual antidote exists in any of the soluble alkaline or earthy sulphates. If none of these is at hand, then the alkaline carbonates, or the bicarbonates,* which are not so irritating as the carbonates. The phosphate of soda is also an excellent antidote. If the patient does not vomit, it will be right also to give an emetic of the sulphate of zinc." (P. 425.)

* We cannot help doubting the propriety of employing the alkaline carbonates in cases of poisoning with the soluble salts of lead.

In the treatment of colica pictonum in its primary stage, every body knows that the conjunction of purgatives and anodynes is the most successful; and mercury to ptyalism has been found useful. In the advanced periods of the disease, when palsy is the chief symptom, the abandonment of the trade, with attention to diet and exercise in the open air, and the use of splints to the hand and forearm, as recommended by Dr. Pemberton, are the appropriate means.

The prophylactic measures are as important as the curative, and of these the essential are strict personal cleanliness; the working clothes should be made not of woollen, but of strong compact linen, which should be changed and washed at least once a week. While at work, a cap of some light impervious material should always be worn. Food should never be taken in the shop, nor before washing, and breakfast should be taken before commencing work in the morning. Constipation should be provided against, and the first indication of derangement of the digestive organs treated with attention. Their diet should be nutritive; and there is some reason for believing that the free use of fat and fatty articles of food is a preservative. The workshops should be spacious, and both thoroughly and systematically ventilated, so as to carry off the floating particles in certain invariable and known courses. The chimneys of smelting furnaces should be high, so as to increase the draught. In white-lead manufactories, the pulverizing has been long performed under water; and at Porto Bello the preparatory process of rolling is also performed entirely under water, or with damping; and, in packing the white lead, the floors are there kept constantly damp; and from these precautions, and great attention to personal cleanliness, the workmen in that manufactory enjoy an immunity from disease unknown in other establishments. (P. 450.)

*Vegetable Acrids.* According to Orfila's experiments, scammony is much less active than jalap. Four drachms of the concrete juice of the root given to a dog produced only diarrhœa, whilst thirty-six grains of the resin of jalap was fatal to a dog in four days. (See experiments of M. Cadet de Gassicourt; Orfila's Toxicol. génèrale, i. 686.)

Savine, although employed with safety and advantage to promote the secretion of pus from blistered surfaces, cannot be applied to a fresh wound without exciting diffuse inflammation. On the effects of oil of savine, Dr. Christison makes the following remarks :

"There is no doubt that, if given in such quantity as to cause violent purging, abortion may ensue; but unless there is naturally a predisposition to miscarriage, the constitutional injury and intestinal irritation required to induce it are so great as to be always attended with great danger, independent of the uterine disorder. In a charge of wilful abortion, the very possession of oil of savine would be a suspicious circumstance, because the notion that it has the power of causing miscarriage is very general and familiar with the vulgar; while, so far as I know, it is not employed for any useful purpose whatever." (P. 453.)

*Animal Acrids.* Cantharides have never been used for the purpose of committing murder; but, on account of its powerful effect on the organs of generation, they have often been given by way of joke, and sometimes for the purpose of procuring abortion. Fatal accidents have been the consequence. Orfila has examined with care the various principles procured by M. Robiquet during his analysis; and it appears to result that the active properties of the fly reside partly in the crystalline principle, and partly in a volatile oil, which is the source of its nauseous odour. The following abstract of a case by M. Biett, of Paris, gives a rational and unexaggerated account of the symptoms produced by cantharides, as they commonly appear. A young man, in consequence of a trick of his companions, took one drachm of the powder: soon afterwards he was seized with a sense of burning in the throat and stomach, and in about an hour with violent pain in the lower belly. When M. Biett saw him, his voice was feeble, breathing laborious, and pulse contracted; and he had excessive thirst, but could not swallow any liquid without unutterable angnish. He was likewise affected with priapism. The pain then became more extensive and severe; tenesmus and strangury were added to the symptoms, and, after violent efforts, he succeeded in passing by the anus and urethra only a few drops of blood. By the use of oily injections into the anus and bladder, together with a variety of other remedies intended to allay the general irritation of the mucous membranes, he was considerably relieved before the second day; but even then he continued to complain of great heat along the whole course of the alimentary canal, occasional priapism, and difficult micturition. For some months he laboured under difficulty of swallowing. (P. 456.)

In a fatal case related in the Gazette de Santé, the brain was found gorged with blood. The omentum, peritoneum, gullet, stomach, intestines, kidneys, ureters, and internal parts of generation, were inflamed; and the mouth and tongue were stripped of the lining membrane.

No antidote has yet been discovered for this poison. Fixed oil, so far from being a remedy, is probably injurious. Oleaginous and demulcent injections into the bladder generally relieve the strangury. The warm bath is an useful auxiliary. Leeches and bloodletting are required according to the degree and stage of inflammation.

Of the species of *Fish* which act deleteriously, either always or in particular circumstances, the muscle is the best known on our coasts. The subject, however, of fish-poison is veiled in great obscurity. Nothing satisfactory has been ascertained as to the signs by which the unwholesomeness in muscles may be ascertained, nor as to its causes. The effects differ in different cases; sometimes they produce symptoms of local irritation only: thus Foderé mentions the case of a sailor at Marseilles, who, in consequence of eating a large dish of them, died in two days, after suffering from vomiting, nausea, pain in the stomach, tenesmus, and quick contracted pulse. The stomach and intestines were found, after death, red and lined with an abundant tough mucus. One of the cases described by Dr. Combe, which however, terminated favorably, is of the same nature. This patient had severe stomach symptoms from the commencement, attended with cramps, and ending in peritonitis, which required the use of the lancet.

But much more frequently the local effects have been trifling, and the prominent symptoms have been almost entirely indirect, and chiefly nervous. Two affections of this kind have been noticed: one is an eruptive disease resembling nettlerash, and accompanied with violent asthma; the other a comatose or paralytic disorder, of a very peculiar description. The former affection has been relieved by ether.

The following summary from Dr. Combe's paper, (Ed. Med. and Surg. Journal, xxix. 88,) gives a correct view of the general phenomena:

"None, so far as I know, complained of any thing peculiar in the smell or taste of the muscles, and none suffered immediately after taking them. In general, an hour or two elapsed, sometimes more; and then the bad effects consisted rather in uneasy feelings and debility than in any distress referrible to the stomach. Some children suffered from eating only two or three; and Robertson, a young and healthy man, only took five or six. In two or three hours they complained of a slight tension of the stomach. One or two had cardialgia, nausea, and vomiting, but these were not general or lasting symptoms. They then complained of a prickly feeling in their hands; heat and constriction of the mouth and throat; difficulty of swallowing and speaking freely; numbness about the mouth, gradually extending to the arms, with great debility of the limbs. The degree of muscular debility varied a good deal, but was a constant symptom. In some, it merely prevented them from walking firmly. While lying in bed, they could move their limbs with tolerable freedom; but, on being raised to the perpendicular posture, they felt their limbs sink under them. Some complained of a bad coppery taste in the mouth, but in general this was an answer to what lawyers call a leading question. There was slight pain of the abdomen, increased on pressure, particularly in the region of the bladder, which suffered variously in its functions. In some the secretion of urine was suspended, in others it was free, but passed with pain and great effort. The action of the heart was feeble, the breathing unaffected; the face pale, expressive of much anxiety; the surface rather cold; the mental faculties unimpaired."

Of the two fatal cases, Dr. Combe says,

"The woman went away as in a gentle sleep, and that, a few minutes before death, she had spoken and swallowed. She died in three hours. The other fatal case was that of a dockyard watchman, who was found dead in his box six or seven hours after he ate the muscles." (P. 465.)

*Venomous Snakes.* A patient of M. Piorry, two hours after being bitten by a viper, had all the constitutional symptoms strongly developed; such as slow, very feeble pulse, nausea, vomiting, and swelling of the face. When a cupping glass was applied for half an hour, the general symptoms ceased, and did not return. Next day diffuse inflammation began, but it was checked by leeches. (P. 472.)

*Of poisoning by diseased and decayed animal matter.* This chapter includes the interesting and important subject of wounds received in dissection. Of the nature of the poison thus introduced nothing is known. It is probable that the developement of ammonia by the process of putrefaction destroys it. It acts like many other animal poisons, sometimes by depressing the vital powers, with little or no inflammation, and requiring a stimulant mode of treatment; sometimes by exciting diffuse inflammation; and occasionally by acting in both ways. Mr. Brodie's paper, in the 57th volume of this Journal; Dr. Duncan's cases, in the Edinburgh Medico-Chir. Transactions, vol. i.; and Mr. Travers' work on Constitutional Irritation, and the masterly review of the latter work in the Edinburgh Med. and Surg. Journal, (xxvi. 340,) contain the most accurate and satisfactory information on the nature and treatment of these cases.

*On the effects of animal matter rendered poisonous by common putrefaction.* Professor Christison remarks, "that, probably in peculiar circumstances, decaying animal matter may excite epidemic fevers:" and this opinion is founded on the researches of MM. Gaspard and Magendie, Leuret and Hamont, and Orfila.* Of animal matter rendered poisonous by modified putrefaction, cheese, bacon, and the German sausage, are the best known examples. "But, from information communicated to me not long ago by Dr. Swanwick," says Dr. C., "an intelligent practitioner of Cheshire, there is some reason to think that a parallel poison is occasionally met with in that country, among the small hill-farms, where the limited extent of the dairies obliges the farmer to keep the curd for several days before a sufficient quantity is accumulated for the larger cheeses." (P. 481.) The symptoms produced by poisonous cheese and sausages are such as result from various degrees of gastro-enteric and gastro-pulmonic inflammations, and subsequently paralysis of the respiratory muscles. The abstract of the prize essay of Dr. Horn, of Berlin, which appeared in the January number of the Edinburgh Med. and Surg. Journal, contained the fullest details on the effects and nature of the sausage-poison presented to British toxicologists; and if Dr. Horn has not succeeded in insulating the poisonous principle, he has shown that the acid of fat is not the *venenum farcimurale*, and that the experiments of Kerner, Westrumb, and Hünefeld are fallacious. The facts stated by him are inconsistent also with the opinion of Dunn, that this poison is the result of putrefaction: at least, in sausages known to be poisonous no sign of putrefaction was perceptible. In vol. 46 of this Journal, an account is given of the work of Kerner on the Sausage poison.

Dr. Christison has introduced into his work a chapter on poisoning by *Mechanical Irritants*, not because such substances have, properly speaking, any poisonous quality, but occasion symptoms, by their mechanical qualities, like those of poisoning; and hence, in a medico-legal work on Poisoning, ought not to be passed over.

The most important are those which cause injury by reason of their roughness, sharpness, or size. The 35th vol. of this Journal may be referred to for an excellent account of the ordinary phenomena in such accidents.

* Journal de Physiol. ii. iii. and vi., and Toxicol. Générale.

Pounded glass is in general certainly not poisonous;* but Mr. Hebb, of Worcester, has related a case (Midland Med. and Surg. Reporter, i. 47,) in which the death of the child resulted probably from this substance inducing gastritis. In another case, described by Portal, he made the patient eat largely of cabbage to envelope the particles of glass, and then gave him an emetic.

The effects of the deglutition of boiling water have been illustrated by an interesting paper of Dr. Marshall Hall, (Med. Chir. Trans. xii.) from which it appears that the disease is cynanche laryngea, terminating fatally by suffocation.

II. Proceeding to the second class, or Narcotic Poisons, we have three important chapters, on opium, hydrocyanic acid, and the poisonous gases.

*Opium*, one of the most important and familiar poisons known, has been frequently used for the purpose of suicide, for the commission of murder, or to induce stupor previous to the perpetration of crimes; and in the practice of medicine has often been the cause of fatal accident.

Passing over the tests of opium, to which subject Prof. C. has added much interesting and original information, and also the physiological and therapeutic effects of moderate doses of opium, and the symptoms of poisoning by opium in man, we proceed to the

*Treatment of poisoning with opium.* The primary object is to remove the poison from the stomach, either by emetics administered in the usual way, by the stomach pump, or by injection of emetics into the veins. By far the best emetic is the sulphate of zinc, in the dose of half a drachm or two scruples, which may be repeated after a short interval, if the first dose fails to act; but, as the poison becomes intimately mixed with the lining mucus of the villous coat, and is never thoroughly removed till the mucus is also removed, the stomach pump appears the preferable mode of emptying the stomach, from the greater facility and certainty with which the mucus may thus be removed.

"The next object in conducting the treatment of poisoning with opium, is to keep the patient constantly roused. This alone is sufficient, when the dose is not very large, and the poison has been discharged by vomiting; and in every case it forms, next to the evacuation of the stomach, the most important part of the treatment." (P. 546.)

This rousing should be effected by constant exercise be-

* For the most satisfactory proof of this, we refer to the experiments of Caldani and Mandruzzatto, in Saggi Scientifici e Litterari dell' Academia di Padova.

tween two men, which should be continued three, six, or twelve hours, or till the stupor is removed. Dashing cold water over the head and breast has been advantageously used, (vide vol. 48, p. 225, of this Journal,) in producing consciousness, and it appears also to be an excellent way to ensure the operation of emetics. Stimulants, such as ammonia, camphor, musk, assafetida, &c., have been in some cases used with advantage. Venesection has been sometimes successfully employed: it ought not to be practised till the stomach has been emptied: after that has been done, if the patient be readily roused, bleeding is not necessary; but, if there be difficulty in producing consciousness, the stomach having been emptied, bleeding will in such circumstances be an appropriate remedy. Artificial respiration is a last resource, and in a few instances has been successfully employed.

"After the breathing has been almost or entirely suspended, the heart continues to beat for some time, and, so long as its contractions continue, there is some hope that life may be preserved. Artificial respiration, we conceive, presents a chance of success in cases where death, if it ensue, will result from paralysis of the respiratory muscles, and where the poison may pass off through the function of respiration.

"There is no useful antidote for opium. Decoction of galls Orfila has found renders it somewhat less active, but his experiments do not assign to it any considerable power. It has been recently stated by Dr. Donné, that iodine, chlorine, and bromiac, are all antidotes for poisoning with vegetable alkaloids, forming compounds with them which are not deleterious. But the delay of ten minutes in the administration of the antidote rendered it useless; and it remains to be proved that the same advantages will be derived from the administration of these antidotes in the instance of poisoning with the crude drug, as with the alkaloids." (P. 663.)

After the opium has been completely removed, the vegetable acids and infusion of coffee have been found useful in reviving the patient, and subsequently in subduing sickness, vomiting, and headach.

*Of poisoning with Hydrocyanic Acid.* This poison is more rapid in its action and more fatal in a minute dose than any other known poison. It is either formed artificially by chemical process, or exists naturally in the leaves, bark, and fruit-kernels of certain plants. The pure acid is liquid, limpid, and colourless. When diffused through the air, the odour has a distant resemblance to that of bitter almonds. It is not the same. It boils at eighty degrees, and freezes at five; it is very inflammable; it decomposes spontaneously, and becomes brown sometimes in an hour, and commonly within twelve hours, unless it is kept very cold. Hence it is very improbable that a case will ever happen in which the medical jurist will have to examine it in its concentrated form. In combination with water, it forms the acid kept in the shops.

The tests for hydrocyanic acid are, its odour, the salts of copper, the salts of the protoxide of iron, and the nitrate of silver. The peculiar odour is a very characteristic and delicate test of its presence.

*Process for mixed fluids.* Dr. Christison commends the process of Lassaigne and Leuret by distillation, which is as follows: The contents (of the stomach), after filtration, are to be neutralized with sulphuric acid, if they are alkaline, in order to fix the ammonia which may have been disengaged by putrefaction; the product is then to be distilled from a vapour bath, till an eighth part has passed over into the receiver, and the distilled fluid is to be tested with the protosulphate of iron in the usual way. Schabarth has objected that the acid may be formed by this process from the decomposition of animal matter. Dr. Christison doubts the soundness of the objection.

*Of the symptoms produced by hydrocyanic acid in man.* Coullon has given a good account of the effects of small doses, as ascertained by experiment on himself. When he took from twenty to eighty-six drops of the diluted acid, he was attacked for a few minutes with nausea, salivation, hurried pulse, weight and pain in the head, succeeded by a feeling of anxiety, which lasted about six hours. Such symptoms are apt to be induced by too large medicinal doses. Another remarkable symptom, which has been sometimes noticed during its medicinal use, is salivation with ulceration of the mouth. Dr. Macleod thrice had occasion to remark this in patients who had been using the drug for about a fortnight, and twice in one individual; and Dr. Granville says he had also twice witnessed the same effect. (P. 563.)

"The most complete account of the symptoms from fatal doses when convulsions occur, is given in a case reported by Hufeland, of a man who, when apprehended for theft, swallowed one ounce of alcoholized acid, containing about forty grains of the pure acid. He was observed immediately to stagger a few steps, and then to sink down without a groan, apparently lifeless. A physician, who instantly saw him, found the pulse gone, and the breathing for some time imperceptible. After a short interval, he made so forcible an expiration that the ribs seemed drawn almost to the spine. The legs and arms then became cold, the eyes prominent, glistening, and quite insensible: after one or two more convulsive expirations, he died, five minutes after swallowing the poison." (564.)

From the observations and experiments recorded, "it is probable that very large doses occasion death in a few seconds; and, at all events, a few minutes will suffice to extinguish life when the dose is considerable; but, if the individual survive thirty or forty minutes, he will very generally recover."

The period within which it begins to operate, is an important question in medical jurisprudence, and the fate of the prisoner on a late trial at Leicester depended on the solution of it. This must depend on the strength of the acid employed. Mr. Macauley, of Leicester, in three experiments, found "that one dog was killed with four drachms in eight seconds; another with four drachms in seven seconds; and another with four and a half drachms in three seconds." We presume the medicinal acid was experimented with.

The proper *treatment* of this form of poisoning is now pretty generally known. "On the whole it appears," says Dr. C. "that the proper treatment of a case of poisoning with hydrocyanic acid consists in the use of the cold affusion, and the inhalation of diluted ammonia or chlorine; and, as chlorine will hardly ever be at hand, ammonia will commonly be employed. Venesection is also probably indicated by the signs of congestion in the head." This is a form of poisoning in which we presume that artificial respiration might be had recourse in desperate circumstances, with a chance of success.

The plants which contain hydrocyanic acid, and whose poisonous qualities depend most probably on its presence, belong to the division *Pomaceæ* of Jussieu's natural family, the *Rosaceæ.* Those which have been most closely examined are the bitter almond, cherry laurel, bird cherry, and peach. The odour which distinguishes these plants is not owing to hydrocyanic acid, for it is equally strong after the acid is thrown down. According to the experiments of Vogel, the volatile oil contains a poisonous principle, independent of the hydrocyanic acid; but we cannot help distrusting the correctness of this opinion.

*On the Poisonous Gases.*

"The subject of the poisonous gases is one of great importance in relation to medical police, as well as medical jurisprudence. They are objects of interest to the medical jurist, because their effects may be, and have been, mistaken for those of criminal violence, and because they have been resorted to for committing suicide. They are interesting as a topic of medical police, since some trades expose workmen to their influence." (P. 583.)

Some gases act negatively on the animal system, by preventing the access of respirable air to the lungs; others are positively poisonous. Nysten* has arranged them according to the results of his experiments on animals, but their effects on man have not been found quite analogous. According to the latter principle, Dr. Christison arranges them into two groups, the irritants and the narcotics. Of the former are nitric oxide gas, nitrous acid vapour, muriatic acid gas, chlorine, ammonia, sulphurous acid, and some others of little consequence.

Under the head of chlorine, Dr. C. remarks "the interesting fact that, during the epidemic fever which raged over Ireland from 1816 to 1819, the people of the manufactory at Belfast were exempt from it." (P. 588.)

The narcotic gases are of much greater importance than the irritants, on account of the singularity of their effects, and the greater frequency of accidents with them. This group includes sulphuretted hydrogen, carburetted hydrogen, carbonic acid, carbonic oxide, nitrous acid, and cyanogen. Dr. Christison has made some interesting extracts from the works of Hallé, (Recherches sur la Nature du Mephitisme des Fosses d'Aisance, 1785,) showing the dangerous accidents arising from the gases of privies, accidents almost peculiar to France from the construction of the fosses; and then notices the fatal cases of cholera (?) which occurred at a school at Clapham last year, and which were attributed to sulphuretted hydrogen from a foul cesspool, the contents of which had been spread over a garden adjoining to the children's playground. Dr. Christison doubts the accuracy of the opinion, and regrets the want of fuller details of the inquiry instituted at the period of the fatal occurrence.

But, of all the deleterious gases, carbonic acid has been the most fruitful source of fatal accidents. Physiologists have doubted whether it is a positive poison, or simply an asphyxiating gas. The facts adduced by Dr. Christison satisfactorily establish the former opinion.

An attempt to inhale pure carbonic acid gas irritates the nostrils and throat so strongly, that the glottis closes, and inspiration becomes impossible. Hence death in this way is purely from suffocation. The effects are very different when the gas is considerably diluted; the symptoms then resemble apoplexy : but these effects differ somewhat according to the source from which the gas is derived, and the

* Recherches Chimico-Physiologiques.

admixtures consequently breathed along with it. Dr. Christison has therefore quoted an example of the effects of the pure gas diluted with air, of the emanations from burning charcoal, tallow, and coal; and, finally, of air vitiated by the breath.

1. "M. Chomel, of Paris, has related a case of poisoning with the gas diluted with air, occurring in the person of a labourer, who was suddenly immersed in it at the bottom of a well, and remained there three quarters of an hour. He was first affected with violent and irregular convulsions of the whole body, and perfect insensibility, afterwards with fits of spasm, like tetanus; and during the second day, when these symptoms had gone off, he continued to be affected with numbness. It is worthy of remark, that, contrary to general belief, these effects may be produced in situations where the air is not sufficiently impure to extinguish lights."

2. Dr. Christison, after giving an abstract of a case by Dr. Babington, (Med. Chir. Trans. i. 83,) of the effects of the fumes of burning charcoal, thus proceeds: "Occasionally the stage of stupor is followed, as in some other varieties of narcotic poisoning, by a stage of delirium, at times of the furious kind, or by a state resembling somnambulism. It does not follow that recovery is certain, because coma has thus given place to delirium." Buchner has related a case which terminated fatally at the end of five days. (P. 688.)

According to the researches of Orfila, the emanations from burning charcoal, when in a state of vivid ignition, consist of carbonic acid gas, as the only foreign ingredient; but, when charcoal burns faintly, fourteen per cent. of carburetted hydrogen may be discovered, and it appears that the vapours are most dangerous in the latter state.

3. The gases produced by the slow combustion of tallow, &c. will produce dangerous symptoms.

"An instance has been recorded in which they proved fatal. A party of iron smiths, who were carousing on a festival day at Leipzig, amused themselves with plaguing a boy, who was asleep in a corner of the room, by holding under his nose the smoke of a candle just extinguished. At first he was roused a little each time; but, when the amusement had been continued for half an hour, he began to breathe laboriously, was then attacked with incessant epileptic convulsions, and died on the third day."

4. "The vapours from burning coal are the most noxious of all kinds of emanations from fuel, and cause peculiar symptoms; but they are less apt to lead to accidents than the vapour of charcoal, as they are much more irritating to the lungs. This effect depends on the sulphurous acid gas which is mingled with the carbonic acid." (P. 600.)

In an accident which happened at Leadhills, in March 1817, from these vapours, four men lost their lives. Of those who recovered, some, when Mr. Braid first saw them, were running about frantic and furious, and striking all who came in their way; some ran off terrified whenever any one approached them; some were singing, some praying, and others lay listless and insensible. Many of them retched and vomited; in some the pulse was quick, in others slow, in many irregular, and in all feeble. All who could describe their complaints had violent headach, some of them tenesmus, and a few diarrhœa. In a few days all recovered except the first four, and three others who had descended to the deeper parts of the mine." (P. 601.) Somewhat analogous to the symptoms now described are the effects of the gradual contamination of air in a confined apartment.

"The treatment of poisoning with carbonic acid consists chiefly in the occasional employment of the cold affusion, and in moderate bloodletting, either from the arm or from the head. In a late case which happened at Paris, where a lady tried to make away with herself by breathing charcoal fumes, and was found in a state of almost hopeless insensibility, various measures were tried unsuccessfully, till cupping from the nape of the neck was resorted to, and she then rapidly recovered." (P. 603.)

Inflating the lungs with oxygen has been found useful in poisoning with carbonic oxide.

III. *On the Narcotico-Acrid Poisons*, Orfila has divided them into six groups. The first of these comprehends the poisons whose principal symptom is delirium. The deadly nightshade, thorn apple, and tobacco, alone have been particularly examined: they all contain alkaloids, to which their virtues are attributable.

"When the extract of Belladonna is rubbed on the skin, vision is not impaired;* though, when it is taken internally, so as to affect the pupils, the sight is commonly much obscured. . . From the cases that have been published, the leading symptoms appear, in the first instance, to be dryness in the throat, then delirium with dilated pupils, and afterwards coma. Convulsions are rare, and when present, slight."

The cases of poisoning which have happened in this country from the Datura Stramonium, have been all accidental; but in Germany, according to a remark which occurs in Gmelin's History of Vegetable Poisons, it has been a good deal employed to produce loss of consciousness and lethargy, preparatory to the commission of various

* Vision is often impaired for some time after the external application of belladonna on the face.—Ed.

atrocious crimes; some of which Gmelin has detailed with edifying particularity. The symptoms in man do not differ essentially from those produced by belladonna.

Emetics and purgatives are the obvious remedies for the poisons just mentioned, and venesection when the determination to the cerebro-spinal system is considerable or continued. The treatment of the poisonous effects of tobacco must be modified, according as the heart or brain is chiefly affected. It appears, from the researches of Parent, Duchatelet, and D'Arcet, that the workmen employed in the tobacco manufactories are as healthy, and live as long, on an average, as other tradesmen.

Passing over the consideration of the poisonous plants of the natural orders *Umbelliferæ* and *Ranunculaceæ*, which present nothing of importance to detain us, we come to the Digitalis Purpurea.

A patient of Dr. Blackall died from the effects of two drachms of the infusion taken daily, but for how many days is not stated. Another fatal case is quoted from six ounces of a strong decoction; and Dr. Christison concludes his account by observing that "the preparations of foxglove are very uncertain in strength. From what I have observed in the course of their medicinal employment, I conceive few powders retain the active properties of the leaves, and even not many tinctures." Our experience of these preparations of foxglove agrees entirely with what is stated by Dr. Christison. We are, however, perfectly satisfied that the Pharmacopœia does not contain a more certain, safe, and efficient medicine than the infusion of the leaves of this plant, when cautiously administered. We have often witnessed its signal benefits in subacute, idiopathic inflammations of the mucous membrane of the lungs, trachea, and larynx; in similar states of disease of the membranes of the brain; in acute rheumatism after early bleeding; in puerperal mania, and other affections. The dose in which we have given it has been half an ounce of the infusion (to an adult) every six or eight hours, watching its effects most closely, and suspending the medicine as soon as sickness or intermission of the pulse occurred. After full experience of the medicine in this way, we can say that we never saw any unfavorable effect produced by it, while its power in arresting certain inflammatory affections appeared to us greater than any other remedy, bleeding excepted.

The therapeutic action of this drug is manifestly owing to its direct action on the heart and arteries, extending to their minutest ramifications. When the medicine is given in smaller doses, and consequently continued during several days before the depressing effect on the heart be produced, the serious consequences of its accumulation in the system may be almost uniformly expected. In the case of a young lady who continued the infusion in the dose of two drachms two or three times a day for several days, alarming symptoms of depression came on, and at the end of six weeks the pulse was slower than natural, and irregular. She entirely recovered, however, and in a few months enjoyed robust health.

The second group of narcotico-acrids consists of those substances which induce violent spasms like tetanus, without impairing sensibility, and prove fatal, for the most part, by suspending respiration. Their poisonous properties are owing to the alkaloids strychnia and brucea, which these plants (the various species of Strychnos and the Brucea antidysenterica) contain.

Except the hydrocyanic acid and some of the poisonous gases, no substance is endowed with such rapid and destructive energy as the strychnia. The effects have been apparent in fifteen seconds, when the substance has been introduced into the cavity of the pleura. Nux vomica, the most familiar species of the strychnos, has been the most ordinary source of the fatal effects of strychnia. "With regard to the dose requisite to prove fatal, the smallest fatal dose of the alcoholic extract yet recorded is three grains." "Hoffmann mentions a fatal case caused by two fifteen-grain doses of the powder; and in Hufeland's Journal there is another, caused by two drachms, which was fatal in two hours." Between two and three drachms of the powder proved fatal in one hour, in a case related by M. Ollier.

Little is known of the treatment in this kind of poisoning. The immediate employment of the stomach-pump or emetics is indispensable. "When nux vomica has been taken in powder, which is the most frequent form in which it has been used, it adheres with great obstinacy to the inside of the stomach. Consequently, whatever means are employed for evacuating the stomach must be continued assiduously for a considerable time. If the patient is not attacked with spasms in two hours, he will generally be safe." (P. 643.) As we have already stated when speaking of morphia, M. Donné believes that iodine, bromine, and chlorine, are antidotes for the alkaloids, and among others for the alkaloid of nux vomica. It is necessary, however, to administer the remedy within ten minutes of the alkaloid; and it has not been ascertained that these antidotes are such to the crude drug.

The symptoms induced by the false Angustura bark are almost the same as those caused by nux vomica. The therapeutic effects of the alkaloid Brucia, as well as Strychnia, have been illustrated by the valuable Hospital Reports of Dr. Bardsley, a review of which has been recently given in this Journal.

We cannot condense the account of the fourth group, the *poisonous Fungi*, which is, as usual, very judicious. One of the best examples of poisoning from this source will be found detailed in vol. 36 of this Journal. Dr. Christison has given additional instances from other sources, and has added an interesting case from Platner, where a girl poisoned her mistress by mixing oxide of arsenic with a dish of mushrooms. The fatal event was at the time attributed to the mushrooms, but the girl subsequently acknowledged the true cause of it.

"The different sorts of grain are subject to certain diseases, in consequence of which, meal or flour made from them is apt to be impregnated with substances more or less injurious to animal life. It is likewise believed that unripe grain possesses properties which render it, to a certain extent, unfit for the food of man." (P. 663.)

The *Secale Cornutum*, or spurred rye, is the most important of the poisons here adverted to. Various causes have been assigned for the production of the spur in rye; and of these, extreme moisture, the growth of a parasitical fungus, a species of scleroticum, according to Decandolle, or puncture by an insect, are the most probable. In confirmation of the latter cause, General Martin Field found that puncturing the glumes of the rye with a needle produced the spur.

A single large dose of the spurred rye (two drachms, for instance,) produces effects analogous to the irritant poisons; but, introduced into the system insidiously, as when taken for some time in rye bread, it occasions, sometimes, a nervous disorder, characterised by violent spasmodic convulsions; at others, a cachectic state of the constitution, terminating in dry gangrene. It does not appear that these two affections are blended together in the same case. The facts presented by Dr. Christison have been chiefly derived from an elaborate essay on the subject by M. Robert, in vol. 25 of Rust's Magazine. As to the medical qualities of the secale cornutum, Dr. C. observes it is the opinion of the best authorities "that it is endowed with the property only of accelerating natural labour, not of inducing it, particularly in the early months of pregnancy." In the fourth Number of the Glasgow Medical Journal, Dr. Macfarlane has given a case in which the ergot appeared to promote the expulsion of the polypus. One drachm was infused in four ounces of water, and one ounce given every two hours. The whole was given before the effect was produced. In the course of a few hours, the tumor was discovered to be in the vagina; four days after the exhibition of the ergot, the polypus fell off. A paper by Dr. Young, in vol. iii. of the Edinb. Med. Chir. Transactions, contains some pretty satisfactory evidence of the power of the spurred rye in accelerating natural labour.

The *Lolium Temulentum*, or darnel grass, is the only poisonous species of the natural order of the grasses. In the 28th volume of this Journal may be found the particulars of some cases of poisoning supposed to be occasioned by the accidental mixture of the lolium with oatmeal. Eighty inmates of the poor-house at Sheffield were attacked with similar symptoms after breakfasting on oatmeal porridge. The chief symptoms were a piercing stare, violent agitation of the limbs, quivering of the lips, frontal headach, confusion of sight, dilated pupil, small tremulous pulse, twitchings of the muscles, and palpitation. In twelve hours all the persons attacked were well but two, who had strong convulsions in the subsequent night, but also eventually recovered.

The chapter on poisoning with *Alcohol* and *Ether* concludes the work, with the exception of the Appendix.

Alcohol or spirituous liquors may prove fatal in the way of coma, or more frequently from a previous excited state of the circulation, causing true apoplexy in a predisposed habit; or still more frequently from the occurrence of some trifling accident, which in his torpid state the individual cannot avoid or remedy, such as exposure to cold, falling with the face in mud or water, suffocation from matters of vomiting getting into the windpipe, &c.

Dr. Alison communicated to Dr. Christison the particulars of a case where death took place from simple intoxication, twenty minutes after the state of lethargy began. On examining the body, Dr. Alison could not discover any morbid appearance, except some watery effusion on the surface of the brain and in the ventricles; but the contents of the stomach had a strong smell of spirits.

When swallowed in large quantity, coma may come on in a few minutes, and become profound as in apoplexy.

"The face is then sometimes livid, more generally ghastly pale; the breathing stertorous, and of a spirituous odour; the pupils sometimes much contracted, more commonly dilated and insensible; and, if relief is not speedily procured, death takes place, generally in a few hours, and sometimes immediately." (P. 678.) Professor Bernt, of Vienna, has quoted four cases, and Dr. Cooke another, where extravasation of blood resulted from poisoning with spirits: it is not, however, an usual result; congestion is far more common. Extravasation is not apt to occur in cases of rapid death, brought on by a very large quantity swallowed at once. The circulation, indeed, in such cases, is in a state quite the reverse of excitement, and accordingly the brain and its membranes are found quite healthy. Dr. Christison is sceptical as to the presence of spirits in the ventricles of the brain, as reported by a few authors. In the well-known case related by Dr. Cooke, it was stated that the liquid was inflammable. "It would have been desirable," says Dr. C. "that Dr. Cooke, or rather his informer, Sir A. Carlisle, had mentioned how the inflammability was proved; for some fallacy may be strongly suspected, because gin of sufficient strength to take fire could not enter the blood-vessels without coagulating the blood, and so preventing its further progress."

Sulphuric Ether is a poison of the same nature as alcohol. "A gentleman, in consequence of inhaling it too long, was attacked with intermitting lethargy for thirty-six hours, depression of spirits, and lowness of pulse." (Journal of Science, iv. 158.)

We here terminate our lengthened analysis of this valuable publication, and if any apology be required for the extended commentary which we now present, the importance of the subjects treated of will readily supply it. To add any thing further to the commendation we passed on the work at the conclusion of our former review would be superfluous, and we have nothing to retract or to modify. Dr. Christison will hereafter be ranked with those illustrious writers on medical jurisprudence, Foderè, Chaussier, and Orfila, who, by their learning, research, and cultivated judgment, have vindicated the claims of that science, which it was the object of their labours to illustrate and extend.

*A Catalogue of the Preparations in the Anatomical Museum of Guy's Hospital; arranged and edited by desire of the Treasurer of the Hospital, and of the Teachers of the Medical and Surgical School.* By THOMAS HODGKIN, M.D. &c., Demonstrator of Morbid Anatomy and Curator of the Museum at Guy's Hospital. 1829.

THE study of anatomy is replete with difficulties, and these arise not only from its intricacy and specific obscurity, but, in this country especially, from the paucity of the materials and means without which it cannot be effectually pursued. The man who would successfully apply his knowledge of anatomy and physiology to the real object of his study, the removal or mitigation of disease, must not rest contented with the mechanical jargon of the schools, sterile lessons of the processes and foramina of bones, the origin and insertion of muscles, the number and distribution of vessels and nerves. He must not be satisfied with the most perfect acquaintance with the descriptive anatomy of authors, nor even with that glossary of knowledge which might qualify him to demonstrate in a dissecting room. It is not by exertions of memory, however laborious, that the mind can be fitted to exercise an art which requires the most comprehensive and careful employ of judgment to connect it effectively with science. Every external sense and every mental power should be accustomed to the contemplation of the structure of the body, and, as far as is known, the individual and united functions of its several parts. The physician should feel the same kind of familiarity with the human machine as the expert musician does with an instrument: and, to attain this, he must know well its common state, the modifications of which it is susceptible, and the manner in which its powers may be variously increased or repressed. We say the same *kind* of familiarity, for the *degree* must ever differ, as one is a comparatively simple, and the other an infinitely complicated, object of study. As it is intricate and complicated, so much the more reason is there for multiplied means of pursuing it; and, next to clinical experience itself, where disease may be studied through all gradations to its triumph in death, we know of no school more instructive than a well-stored and scientifically arranged anatomical and pathological museum. But such a museum is not an infinite collection of musty preparations, remarkable rather for deviations from nature, both original and produced by time, than for illustrating ordinary courses of disease; all arranged as artificially as the most rigid disciple of Linnæus might desire: a tattoed head, a monstrous conception, and a necrosed bone, ranged together by way of pleasing variety. Such we have seen, and such may do for the vulgar to gaze at, and for the collector complacently to look over, as so many valuable *curiosities*, and monuments of his cumulating labour. An anatomical museum, to instruct the mind, should, as much as possible, represent the ordinary course of nature, in the production, state, and alteration of parts. The mechanism of the frame, it is true, requires many artificial processes of analysis to acquaint us with its structure and use; but, this knowledge attained, the mind cannot too much accustom itself to view all the powers in combination and acting in concert as one whole, rather than in systems or parts. It is in such combination and concert that the living body presents its properties; and, to an oversight of this, and a too partial view of organization and its elements, we would ascribe the multitude of false theories and erroneous opinions which, as phantoms or false lights, have continually beguiled the student from the path of true knowledge.

It is our pleasing duty to notice the institution of a museum, which, if it go on to maturity as its infancy has begun, bids fair in a few years to equal in extent, and far to surpass in utility, any similar establishment in the kingdom. We know of no instance of zeal for the true interests of the science more creditably or more successfully exerted, than in the formation and philosophical arrangement of the museum at Guy's Hospital, which, in the course of four or five years, has been brought to its present forward and instructive condition. To the benevolent and enlightened zeal of Benjamin Harrison, Esq. treasurer to Guy's Hospital, this institution owes its birth; and when we express our unqualified and grateful praise of the direction and activity of his exertions in the promotion of our science, we know that we shall be joined by every sincere cultivator and well-wisher of our profession. It is not a slight proof of the judgment and discernment of Mr. Harrison, and of the governors of the hospital, that they selected as curator of the museum the able and indefatigable writer of the Catalogue which now lies before us.

To write a Catalogue may be thought a work of labour, rather than of genius or judgment; but, when it is recollected that an arrangement or classification is required to render the Catalogue fit for its purpose, it must appear that a philosophic mind can alone be fitted for the task. Dr. Hodgkin has, we think, judiciously classed the preparations of both special and pathological anatomy according to the organs or systems of apparatus. This method we prefer to that of elementary tissues, since, however philosophical the latter may be, it still involves many doubtful points, and is not so immediately applicable to practical study as the organic classification.

The Catalogue is divided into three parts. The first comprehends the objects of special anatomy; the second, pathology; and the third, zoology and comparative anatomy. The two first of these include the following sections: 1. Bones; commencing by the vertebral column, as the most essential part of the skeleton, the characteristic of that class of which man is the head. 2. Soft parts about the bones. 3. Vascular, or circulatory systems. 4. Nervous system, and organs of the senses. 5. Vocal and respiratory organs. 6. Digestive organs. 7. Urinary organs. 8. Genital organs of the female. 9. Genital organs of the male. 10. Peritoneum, and (in Part II.) specimens illustrative of hernia. 11. Preparations relating to conception and utero-gestation. 12. Parasytical animals.

The arrangement of Part II. is further regulated according to the nature of the deviation from the normal state, as caused by diminution or increase of the natural function, its various modification, &c.; and thus an additional help is given to the study of the preparations in the museum, which, by means of the Catalogue, the student can himself classify and understand. To most of the sections the author has prefixed introductory remarks; and these exhibit such an acquaintance with the latest writers, as well as talent for original observation and thought, as well entitle them to the perusal of the profession at large. Dr. H. has not given dry extracts from works of anatomy; but, in an interesting and instructive manner, he points out to the student the prominent features of the later opinions and discoveries of anatomists and pathologists, and, with much modesty and judgment, offers his own views respecting them. He has himself been an industrious and zealous observer; and he has enriched the Catalogue with many valuable results of his labours. Our limits will not permit us to do more than glance through the pages of prefatory observations scattered through the Catalogue, and which, if collected and diluted in the ordinary bookmaking fashion, might make a goodly volume.

Before Section i. the author gives a sketch of that part of the systems of unity of formation that applies to the bones of the head, which these suppose to be nothing else than modified vertebræ. The following remarks are added, and they may be presented as a fair sample of the style and matter of the text.

"Although the existence of a certain analogy between the bones of the cranium and the vertebræ, not merely in their use, but in their structure, must be admitted by all who will carefully examine the subject, various objections suggest themselves with reference to most of the modes in which it has been attempted to exhibit the application of the principle. It will not be necessary here to do more than offer a few remarks on the system just described, as the result of the labours of Geoffroy St. Hilaire.

"In the first place, the Professor's mode of reasoning seems to be not altogether exempt from this important defect, that many of the steps of his argument want the support of proof. The ingenious theory of the formation of the vertebræ, originally composed of nine primitive portions, appears to be precisely in this predicament; since, though it may be rendered plausible in one or more particular vertebræ, it is by no means the case with others, whatever be the period of formation at which the examination was made. But, were this point to be conceded to the plea of our inability properly to make the examination of parts so minute and tender as those in question must be in the youngest embryo, a new difficulty meets us in the very next step; since, according to the Professor's own statement, the developement of one or other of the rings or arches attached to the body may acquire an extraordinary developement at the expense of the opposite circle, which, in consequence, is either wholly or partially lost. Hence, on the hypothesis that the cranium is composed of developed vertebræ, it is by no means necessary that the number of its component parts should be an exact multiple of nine. Again, by admitting into the list of bones, parts which are never met with but in form of cartilage, such as the tarsi and septum narium, a wide door is opened to doubt, not to say error. It is this doubt which, *à priori*, induces a suspicion of the correctness of the calculation by which it is attempted to be shewn that seven vertebræ are to be sought among the elements of the skull. Let the facts be examined, and it will probably be concluded, *à posteriori*, that three or four of the supposed anterior vertebræ must be discarded, and the number of primitive sections, or cinctures, analogous to vertebræ, reduced. It is in their important office of supporting and protecting a portion of the central part of the nervous system, by means of an arch or ring fixed upon a body, which, united to its fellows, concurs to form a medial support to the bony framework of the animal, that the bones of the cranium are in some degree analogous to those of the spine. Now, the bones of the face can scarcely be said to participate in these resemblances. Like those of the extremities, they are subservient to functions in which the nerves, or, in other words, the branches proceeding from the centre of the nervous system, rather than this centre itself, are directly concerned. Though more or less closely brought together upon the median line, they are not therefore necessarily to be considered as the continuation of the central stem, either in function or structure. Were the nerves of smell, instead of being directed to a single organ on the median line, to be distributed to two symmetrical organs more or less widely separated from each other, as is the case perhaps in some insects, we should no more think of seeking in the elements of the nose for the repetition of the mode of formation proper to the vertebræ, than we are disposed to do in those cases of monstrosity in which the lower or posterior extremities happen to be united, so as to constitute a sort of tail. It is unnecessary on the present occasion to push the inquiry further, or to multiply facts, which might be adduced for its illustration. What has been said proceeds from no wish to disparage the principle, but is designed rather to stimulate to its legitimate investigation, and to point out the danger which those incur who are directed in their investigations by the desire of establishing a preconceived hypothesis."

In a page prefatory to section ii. the author notices Meckel's and Home's observations of the globular structure of the fibres of muscles, and brings forward his own and Mr. Lister's examination with the aid of an achromatic microscope of superior power, belonging to the latter gentleman, in opposition to them. They thus discovered linear fibres as far as the magnifying power could reach; and certain transverse striæ or lines, crossing these at right angles, gives a reticulated appearance, which the instruments used by former observers might have represented as globular. On this we suspend our judgment. The result of our own observations, carefully made some years ago, were decidedly confirmatory of the opinions of Home, Prevost and Dumas, and others, and consequently in opposition to that now before us.

The middle coat of the arteries, Dr. H. observes (before section iii.) is merely fibrous, and wants the transverse striæ. The question of absorption is touched upon; and the author advances a curious idea, that the venous and lymphatic systems are subservient to a process of separation in their powers of absorption: "that whilst the lymphatic vessels act more particularly on those fluids which possess an alkaline tendency, the veins, on the other hand, admit the acids and substances allied to them." We confess that this view seems to us too fanciful ; and, if it be not refuted by the fact that the venous blood is itself alkaline, we conceive it would be difficult to prove in any instance that it is ever distinctly acid.

Some original observations on the structure of the brain and nerves are presented in the fourth section. In no part of these could the author discover any globular structure; nor is there, as is generally supposed, any medullary matter in the nerves. An extract from an unpublished memoir of M. Foville, of Rouen, on this subject, is subjoined.

Sect. xi. Part i. contains preparations illustrative of the fluids of the body, and their diseases. The microscopical examination of the blood by our author and Mr. Lister is detailed, and, if it be accurate, presents a view of the constitution of the red particles very different from those of the latest observers. They describe them as "circular, flattened, transparent cakes, which, when seen singly, appear to be nearly or quite colourless. Their edges are rounded, and, being the thickest part, occasion a depression in the middle, which exists on both surfaces." They could never detect any central colourless globule, as described by Home, and Prevost and Dumas. Of course, such observations, opposed as they are to those which have preceded, require strong confirmation before they can be admitted.

The second Part, comprising the preparations of morbid anatomy, is enriched by the short descriptions added to each preparation, with many references to the hospital books, and other sources of more extended detail of the cases from which they were derived. These are their best comment; and, without such information, we think specimens of morbid anatomy little better than pieces of curiosity.

Dr. H. introduces the fifth section by some interesting remarks and extracts respecting goître and cretinism. He very properly rejects the opinion that the drinking of snow water is a cause of goître. It is very strange that such an hypothesis should ever have prevailed. Descending from the valley of Chamouix, by the valley of the Trient and the Forclâz, we asked our guide how it was that the inhabitants of these high regions never had goître? "O!" said he, "that is *because they drink snow water*: it is those that live in the Vallais, and drink *les eaux des marais*, that are goitreux. Be this as it may, it is certain that, instead of prevailing more, goître nearly or entirely disappears in all those elevated situations, which are most directly supplied by snow water. Dr. H. says,

"I confess that I am inclined to accuse the water; but surely not because it once existed in the solid form. The salts of lime appear a much more likely cause. Of the water in those parts of South America in which bronchocele occurs, I know nothing; but I have seen goître in an evidently calcareous district in Normandy.

Chalk abounds in Sussex, and limestone in Derbyshire and Yorkshire. Goître has at various times appeared amongst the children at a large school in the last-mentioned county, and disappeared at intervals; and those changes have coincided with circumstances affecting the water used as drink by the children. Sometimes it was rain water, when the goître ceased; sometimes from one spring, sometimes from another."

We cannot fall into our ingenious author's opinion without far more convincing evidence than we have yet seen adduced. That a calcareous impregnation in the water used for drink may be one of the causes that contribute to produce goître, we are not disposed to deny; but that it is the only cause, we think disproved by the fact that, in many calcareous districts, where the water so abounds in carbonate and sulphate of lime that culinary utensils become furred by the deposit, and muriate of barytes produces an abundant precipitate, this affection rarely or never occurs. Thus it is in the more open and level parts on and about Salisbury plain, the substratum of which is chalk, of all forms of carbonate of lime the most liable to solution. We are more inclined to accuse *the air;* and whether it be by humidity, or any other state arising from terrestrial exhalations, we do not see how the situations in which this disease is most generally produced, can operate through any other medium. We believe that goître rarely occurs in a flat or open district; and, in mountainous countries, it is in the lowest and deepest valleys, gorges so abruptly walled, or whose sides are so covered with luxuriant vegetation that the sun's rays and purifying winds can but imperfectly enter, the margins of stagnant water and damp marshy bottoms, that are the most remarkable, if not the only, places of its occurrence. The higher valleys, however narrow and secluded they may be, are preserved from the excessive accumulation of humidity by the greater scantiness of vegetation, and the freer evaporation under diminished atmospheric pressure. These remarks agree with, and in fact have arisen from, many observations which we have personally made both in Switzerland and in Britain.

We cannot agree with Dr. H. in entirely separating bronchocele from cretinism. That goître often occurs without any of the disgusting characteristics of the miserable cretin, is quite certain; but we believe that cretinism is never met with where goître is not also to be found. The valley of the Rhone above the lake of Geneva, particularly the neighbourhood of Martigny, presents the most frequent examples of cretinism; and in these situations there seems to be an excess of those causes to which we have attributed the production of goître.

There are some interesting remarks prefixed to the sixth section, on the structure of the liver, and the author directs the attention of pathologists to the manner in which the acini, and the interstitial structure respectively, become affected by disease. It is on the increase of the acini that most cases of enlargement of the liver depend; and it is in these also that the fatty degeneration takes place. It is rather singular that so few cases of this disease should have been met with in England; since Louis remarked it in a third of his phthisical patients.

We must pass over the observations introductory to the remaining sections; not from their want of interest, for some are very curious, as, for example, those on conception and monstrosities; but that we conceive we have done enough to show the merit of the author in the composition of this valuable Catalogue, and in the arrangement of the Museum. The wax preparations we have examined with attention and admiration: they are, indeed, the pride of the Museum, and we were most agreeably surprised to find that such beautifully exact representations of disease had come from the hand of a native artist, Mr. JOSEPH TOWNE. Cutaneous diseases are thus represented far more strikingly and truly than could be done by the most highly-wrought paintings; and we congratulate the teachers of the School of Guy's Hospital in having at their command such means of conveying instruction, as no other lecturers in this country possess.

We conclude by most heartily wishing success to the institution; and we think that Dr. Hodgkin has conferred a real benefit both on the School of Guy's Hospital, and on the profession at large, by the patient and zealous labour which we have discovered in his work.

